# A C-terminal ataxin-2 disordered region promotes Huntingtin protein aggregation and neurodegeneration in Drosophila models of Huntington’s disease

**DOI:** 10.1093/g3journal/jkab355

**Published:** 2021-10-09

**Authors:** Joern Huelsmeier, Emily Walker, Baskar Bakthavachalu, Mani Ramaswami

**Affiliations:** 1 School of Genetics and Microbiology, Trinity College Institute of Neuroscience, Trinity College Dublin, Dublin 2, Ireland; 2 School of Basic Science, Indian Institute of Technology, Mandi, Suran 175075, India; 3 National Centre for Biological Sciences, TIFR, Bangalore 560065, India

**Keywords:** Ataxin-2, Htt, neurodegeneration, RNP granule, *Drosophila*

## Abstract

The Ataxin-2 (Atx2) protein contributes to the progression of neurodegenerative phenotypes in animal models of amyotrophic lateral sclerosis (ALS), type 2 spinocerebellar ataxia (SCA-2), Parkinson’s disease, and Huntington’s disease (HD). However, because the Atx2 protein contains multiple separable activities, deeper understanding requires experiments to address the exact mechanisms by which Atx2 modulates neurodegeneration (ND) progression. Recent work on two ALS models, C9ORF72 and FUS, in *Drosophila* has shown that a C-terminal intrinsically disordered region (cIDR) of Atx2 protein, required for assembly of ribonucleoprotein (RNP) granules, is essential for the progression of neurodegenerative phenotypes as well as for accumulation of protein inclusions associated with these ALS models. Here, we show that the Atx2-cIDR also similarly contributes to the progression of degenerative phenotypes and accumulation of Huntingtin protein aggregates in *Drosophila* models of HD. Because Huntingtin is not an established component of RNP granules, these observations support a recently hypothesized, unexpected protein-handling function for RNP granules, which could contribute to the progression of Huntington’s disease and, potentially, other proteinopathies.

## Introduction

Neurodegenerative diseases (ND) differ in clinical pathologies. For instance, amyotrophic lateral sclerosis (ALS) primarily causes the degeneration of motor neurons, spinocerebellar ataxias primarily affect cerebellar Purkinje cells and Huntington’s disease affects cortical and striatal neurons (Vonsattel *et al.* 1985; Seidel *et al.* 2012; Braak *et al.* 2013). However, the presence of pathological protein aggregates, nuclear, cytoplasmic or even extracellular, is a shared feature of most ND (Ross and Poirier 2004; Ramaswami *et al.* 2013; Soto and Pritzkow 2018). Most notably: extracellular Amyloid-ß (A-ß) plaques as well as neurofibrillary tangles of hyper-phosphorylated Tau protein occur in Alzheimer’s disease patients (Duyckaerts *et al.* 2009; Peng *et al.* 2020); prion protein filaments are found in prion diseases (Prusiner 1991); and α-Synuclein and Ubiquitin containing inclusions called Lewy bodies (LB) occur in Parkinson’s disease (Polymeropoulos *et al.* 1997; Spillantini *et al.* 1997; Luk *et al.* 2012; Henderson *et al.* 2019). Similarly, the Spinocerebellar ataxias (SCAs) caused by expansions of intrinsic poly-glutamine tracts in the causative proteins, are associated with the presence of respective mutant-protein aggregates, particularly in neurons that lie within vulnerable brain regions (Lastres-Becker *et al.* 2008; Seidel *et al.* 2012; Rüb *et al.* 2013). The intracellular location of such inclusions can vary: for instance, in SCA-3, neuronal inclusions of Ataxin-3 are mostly nuclear, while in SCA-2 inclusions of Ataxin-2 are mainly cytoplasmic (Huynh *et al.* 2000; Seidel *et al.* 2017).

A causal role of these usually amyloid-rich intracellular aggregates in disease is argued by several lines of evidence. For Alzheimer’s disease, striking genetic analyses have shown that human mutations in APP that promote the production of A-ß also promote Alzheimer’s disease and mutations that specifically reduce A-ß production confer resistance (Jonsson *et al.* 2012). A common pathway by which such aggregates potentially cause disease, is suggested by increased levels of integrated stress response (ISR) signaling in animal models of neurodegenerative disease (Moreno *et al.* 2012). Elevated levels of misfolded proteins results in activation of stress kinases, which by phosphorylating eIF2a reduce translation efficiency (Harding *et al.* 2003; Wek *et al.* 2006; Pavitt 2018). Consistent with a causal role in disease progression, restoring protein translation by blocking eIF2a phosphorylation or its consequences can delay, block or even reverse disease progression in animal models (Moreno *et al.* 2012; Halliday *et al.* 2015, 2017; Sidrauski *et al.* 2015). Therefore, although definitive clinical support for the “amyloid hypothesis,” that amyloid aggregates play major causal roles in disease, remains elusive, there is considerable interest in therapeutic strategies based on either inhibiting ISR signaling or reducing the formation of disease-specific protein inclusions (Duennwald and Lindquist 2008; Shacham *et al.* 2019; Ganz *et al.* 2020).

Three main lines of evidence implicate ribonucleoprotein (RNP) granules in the formation of disease-associated protein aggregates in ALS and Frontotemporal Dementia (FTD). First, several RNP granule components including TDP-43 are enriched in ALS/FTD associated neuronal inclusions (Hart *et al.* 2012; Kim *et al.* 2013; Gopal *et al.* 2017; Markmiller *et al.* 2018). Second, mutations in different RNP granule components have been found in ALS/FTD patients (Elden *et al.* 2010; Ramaswami *et al.* 2013; Taylor *et al.* 2016). Third, many pathogenic mutations occur in assembly-prone intrinsically disordered regions (IDRs) commonly found in RNA-binding proteins and act to promote aggregate formation (Guo *et al.* 2011; Kim *et al.* 2013).

Several analyses have shown that the RNP-granule protein Ataxin-2 has multiple functions, being required for miRNA activity, translational repression, translational activation, mRNA polyadenylation, and RNP-granule assembly (Nonhoff *et al.* 2007; Mccann *et al.* 2011; Lim and Allada 2013; Zhang *et al.* 2013; Sudhakaran *et al.* 2014; Bakthavachalu *et al.* 2018; Inagaki *et al.* 2020). Ataxin-2 protein is also required for pathological aggregate formation and neurodegeneration in animal models of ALS/FTD (Elden *et al.* 2010). Thus, knockdown of Ataxin-2 via antisense oligonucleotides delays the maturation and recruitment of TDP-43 to RNP granules (stress granules) *in vitro*, reduces pathologies and extends lifespans in a TDP-43 mouse model of ALS (Becker *et al.* 2017; Scoles *et al.* 2017). Studies in *Drosophila* have clarified that of Ataxin-2’s multiple activities, its specific role in RNP-granule assembly contributes to the progression of neurodegenerative phenotypes in fly models of ALS/FTD (Elden *et al.* 2010; Bakthavachalu *et al.* 2018). Thus, genetic deletion of a C-terminal intrinsically disordered region (cIDR) required for efficient formation of RNP granules results in perfectly viable adult flies, which, remarkably, show resistance to cytotoxicity in two *Drosophila* ALS models, of C9ORF72 and FUS, respectively (Bakthavachalu *et al.* 2018). Taken together with additional observations that the Atx2-cIDR is also required for the aggregation of FUS protein and C9-associated dipeptide repeats in cell culture, these results support a model in which RNP granules, perhaps by concentrating aggregation-prone proteins within membrane-less organelles, enhance the efficiency with which initial pathogenic aggregates associated with ALS/FTS can be nucleated (Becker and Gitler 2018).

A more recent study of Huntington’s disease (HD) in *Drosophila* showed a connection between polyQ expanded Huntingtin (Htt) and Ataxin-2 (Xu *et al.* 2019b). In Huntington’s disease, pathogenic expansions occur within an endogenous polyQ domain starting at amino acid 17 of Htt. Via proteolytic processing and aberrant splicing, an N-terminal region of the Htt protein is separated from the full-length protein. This fragment containing expanded poly-Q domains is found in cellular aggregates (Landles *et al.* 2010; Sathasivam *et al.* 2013; Peskett *et al.* 2018). Expression of pathogenic forms of Htt-polyQ in *Drosophila* resulted in the formation of Htt-polyQ aggregates most easily visualized in cells of the eye imaginal disc, death of adult photoreceptors or, if appropriately expressed, in death of cells that control the circadian clock in *Drosophila* (Jackson *et al.* 1998; Romero *et al.* 2008; Weiss *et al.* 2012; Schilling *et al.* 2019; Xu *et al.* 2019b). Remarkably RNAi mediated knockdown of Atx2, in clock cells expressing Htt-polyQ, reduced both Htt-polyQ aggregate formation and cytotoxicity in these cells (Xu *et al.* 2019b).

Given that Htt is not an established component of neuronal RNP granules, we were interested in examining whether Atx2’s RNP-granule forming ability was also required for its role in promoting Htt-polyQ induced protein aggregation and neurodegeneration. We report that removal of the Atx-2 cIDR not only protects against photoreceptor degeneration in two *Drosophila* HD models but also greatly reduces the Htt-polyQ aggregate size and number in eye imaginal disc cells. We document these observations, which are consistent with recent reports indicating overlapping pathways and similarities between ALS and HD cellular pathologies (Freibaum *et al.* 2015; Zhang *et al.* 2015; Grima *et al.* 2017). We discuss why these results may support a model in which RNP granules serve a secondary general function in sequestration of misfolded cellular proteins (Al-Ramahi *et al.* 2007; Lessing and Bonini 2008; Ganassi *et al.* 2016; Mateju *et al.* 2017).

## Materials and methods

### Drosophila husbandry and fly stocks used

*Drosophila* stocks were maintained at 25°C in cornmeal agar. Experimental crosses were carried out at 25°C and 60% humidity with a 12 h light-dark cycle. The *GMR-Gal4* (BDSC 1104) and *UAS-htt-ex1-Q97* (BDSC 68417) lines were obtained from the Bloomington Drosophila Stock Centre (Indiana). *UAS-mRFP-mHtt-Q138* and *UAS-mRFP-mHtt-15Q* were a gift from Troy Littleton (Weiss *et al.* 2012). *UAS-TDP-43-wt* and *UAS-TDP-43-M337V* were a gift from Paul Taylor (Ritson *et al.* 2010). *atx2-ΔcIDR-GFP* was previously published (Bakthavachalu *et al.* 2018).

### Immunofluorescence and confocal imaging

Wandering 3rd instar larvae were collected from respective genetic crosses and eye imaginal discs were processed for immunostaining as previously described (Sudhakaran *et al.* 2014). Each immunostaining experiment was reproduced in at least three independent biological replicates: for each replicate, discs dissected from multiple larvae were individually examined.

Primary antibodies used were: rabbit anti-RFP (Invitrogen R103367,) at 1:1000, rabbit-anti-Rasputin (Aguilera-Gomez *et al.* 2017) 1:1000, rabbit-anti-Caprin (Papoulas *et al.* 2010) at 1:1000, rat-anti-Elav-7E8A10 (Developmental Studies Hybridoma Bank) 1:300 and chicken-anti-Ataxin-2 (Bakthavachalu *et al.* 2018) at 1:500. Secondary antibodies were used at 1:1000 dilution: Alexa Fluor^®^488 goat anti-Chicken (Invitrogen A11039), Alexa Fluor^®^488 goat anti-Rabbit (Invitrogen A11078), Alexa Fluor^®^488 goat anti-Rat (Invitrogen A11006), and Fluor^®^555 goat anti-Rabbit (Invitrogen A21428).

Prepared eye-imaginal discs were mounted in Vectashield Mounting Medium (Vector Labs, H-1000) on microscope slides and imaged on a Zeiss LSM880 confocal microscope.

### Eye degeneration

To assess eye degeneration *GMR-Gal4* was to respective UAS-effector lines to appropriately model degenerative disease. To examine the role of the Atx2-cIDR in promoting degeneration in these contexts, double-balanced *GMR-Gal4*; *atx2-ΔcIDR-GFP* flies were crossed to UAS-effector lines that either included *atx2-ΔcIDR-GFP*, if the effector transgene (*e.g.*, UAS-Htt-138Q-RFP) was on the second chromosome, or recombined with *atx2-ΔcIDR-GFP* if the effector transgene (*e.g.*, *UAS-mRFP-mHtt-Q138*, *UAS-mRFP-mHtt-15Q*, *UAS-htt-ex1-Q97*) was on the third chromosome.

Fly eyes were scored within 24 h of eclosion and every 10 days thereafter until day 50. Eye phenotypes were scored into 3 categories. Normal eyes that did not have any discoloration or visible roughness. Mild phenotypes represent eyes that have local or wide spread discoloration, roughness, or glossiness without necrotic areas. Strong phenotypes represent eyes that additionally exhibit necrotic areas identified by black spots.

**Figure 1:**
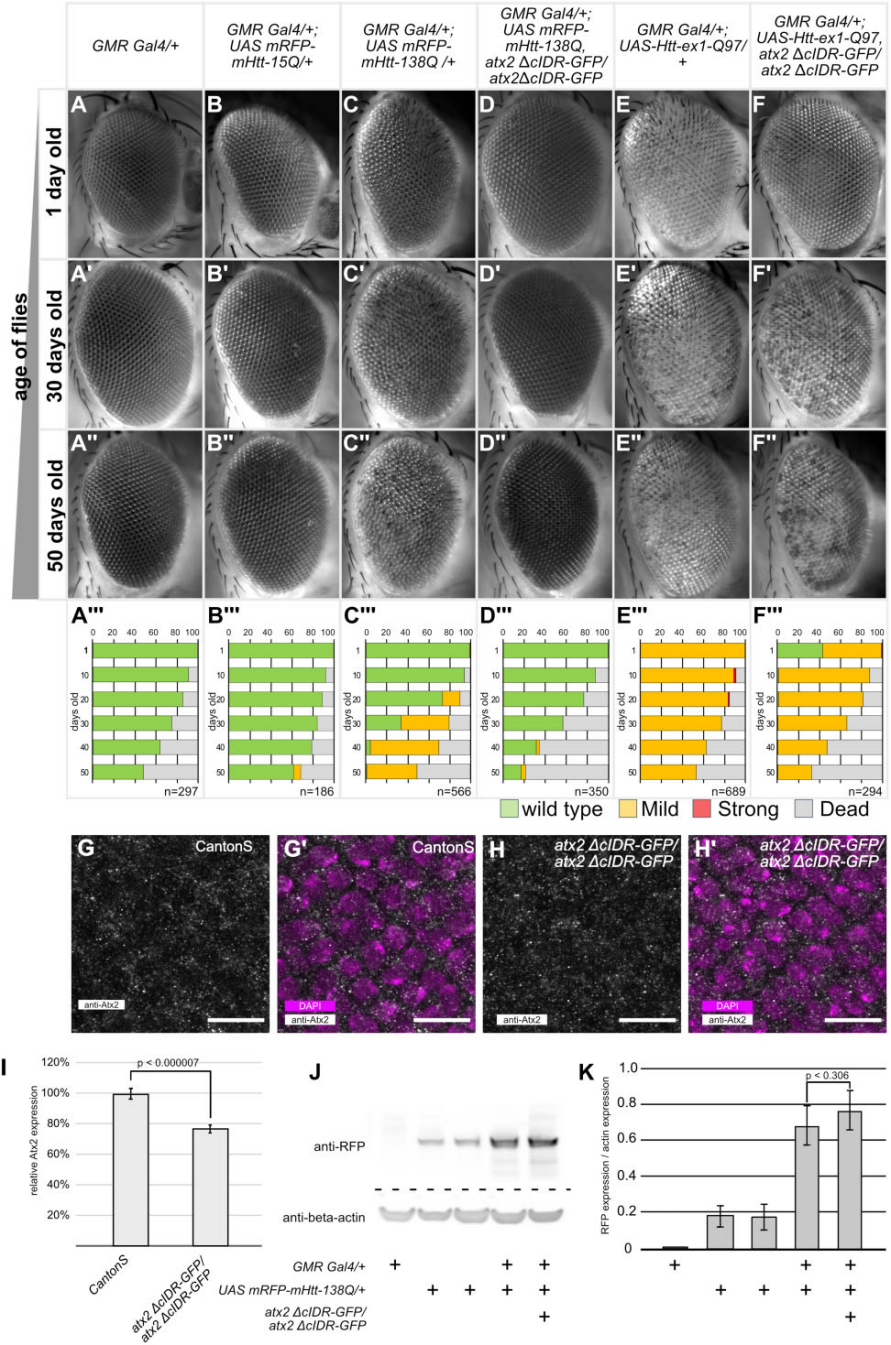
Atx2-cIDR-GFP is required for Htt-polyQ induced neurodegeneration. (A–F’’) Representative images of *Drosophila* eyes taken at the indicated time points (1 day, 30 day, 50 day old, and female flies). Genotypes are indicated at the top of each column. Inserts are quantifications of phenotypes for the corresponding genotype and age (male and female flies). (A’’’–F’’’) quantification of phenotypes observed at 10-day intervals between 1 and 50 days after eclosion. Green bars indicate percentages of wild-type eyes, yellow bars a mild phenotype with patches of discoloration or rough surface visible, red bars are eyes with necrotic spots and gray bars percentage of dead flies. Number (*n*) for each genotype at day 1 is indicated in the insert. (G–H’) Confocal images of eye imaginal discs of 3rd instar *Drosophila* larvae, posterior is up. (G/H) Tissue stained with anti-Atx2 antibody (gray). (G’/H’) Tissue stained with anti-Atx2 antibody (gray) and DAPI (magenta). G/G’) Wild-type Canton S, H/H’) homozygous *atx2 ΔcIDR-GFP*. Sub cellular localization of wild-type Atx2 is similar to Atx2-*Δ*cIDR-GFP. Scale bars 10 μm. (I) Quantification of fluorescence intensity of Atx2 antibody reactivity, normalized to fluorescence intensity in CantonS. Three biological replicates per genotype, at least 5 discs were analyzed per replicate. Expression levels of *atx2 ΔcIDR-GFP* are significantly lower than wild-type Atx2 (2-tailed Student’s *t*-Test, *P* = 0.0000069). Error bars SEM. (J) Representative Western blot of head lysates from 1 day old flies, 10 heads per lane. Genotypes are indicated below image. Immuno-reactivity to anti-RFP (top) and anti-beta-actin (bottom). (K) Quantification of the expression levels of *UASmRFP-mHtt-138Q* as measured by Western-Blot immune-reactivity to anti-RFP. mRFP-mHTT expression levels are normalized to the corresponding beta-actin loading control. Three independent replicates were carried out. Genotypes are indicated below the bar graph. Expression levels of mRFP-mHtt-138Q in *GMR-Gal4/+; UAS-mRFP-mHtt-138Q*, *atx2-ΔcIDR-GFP/atx2-ΔcIDR-GFP* are not significantly different from the levels in *GMR-Gal4/+; UAS-mRFP-mHtt-138Q* flies (one-tailed Student *t*-test, *P* = 0.305). Error bars are SEM.

The number of flies scored per genotype are listed in Figure 1, A–F and Supplementary Figure S2, A’’–E’’. Bar graphs in the panels of Figure 1, A’’’–F’’’ and Supplementary Figure S2, A’’–E’’ represent the percentage of flies corresponding to each phenotype and dead flies at the respective day. Both male and female flies were examined; for logistical reasons, scoring was not performed blind to genotype.

### Eye image acquisition

In addition to flies used to score phenotypes, a small batch of flies was aged separately. Flies of this cohort were sacrificed and used for image-acquisition. At specific ages flies were transferred into a 1.5 mL micro-centrifuge tube and frozen at 20°C. For imaging, flies were pinned with insect needles to a Sylgard plate. In order to image the whole compound eye, flies were oriented with one eye clearly visible. Automated Z-stack images were taken on a Zeiss AxioImager Z1 with a 10x lens, with a stack distance of 18 μm. To generate the final images, image-stacks were “focus stacked” using ImageJ (Schneider *et al.* 2012), followed by orientation and cropping of the images. In all images, anterior is to the right, dorsal to the top.

### Protein extraction from *Drosophila* heads

For each genotype 40 fly heads of 1-day-old flies were collected on ice, and transferred into a microcentrifuge tube containing pre-cooled 40 μL extraction buffer (20 mM HEPES, pH 7.5, 100 mM KCl, 5% glycerol, 10 mM EDTA, 0.1% Triton, 1 mM dithiothreitol, 0.5 mM phenylmethylsulfonyl fluoride, 20 mg/mL aprotinin, 5 mg/mL leupeptin, 5 mg/mL pepstatin A). Heads were ground on ice with a pestle for 30 s followed by brief centrifugation at 4°C. This was repeated three times. After the last repeat samples were centrifuged for 5 min at 4°C, the supernatant was collected into a new microcentrifuge tube. The volume of each tube was adjusted to 40 μL with Extraction buffer. Four microliters of 10x Sample Reduction Agent (Invitrogen, B0009) and 10 μL 4x LDS Sample Buffer (Invitrogen B0007) were added to the samples and heated for to 70°C for 10 min. Samples were then stored at −80°C until use.

*Drosophila* head lysates, corresponding to 10 heads per sample, were separated by SDS gel electrophoresis and subsequently transferred using the iBlot 2 System (Invitrogen, IB21001S) according to the manufacturer’s instructions. Western blots were performed using rabbit anti-RFP (abcam ab62341, 1:5000) and mouse anti-beta-actin (Proteintech 6008-1, 1:5000). Secondary antibodies conjugated with HRP from Jackson Immunoresearch were used at 1:10,000 dilution.

### Image analysis

Western blot images were analyzed using the gel densitometry tool in ImageJ (Schneider *et al.* 2012). Expression of mRFP-mHtt-Q138 was calculated in relation to the expression of the beta-actin loading control. The experiment was independently repeated in triplicate.

**Figure 2:**
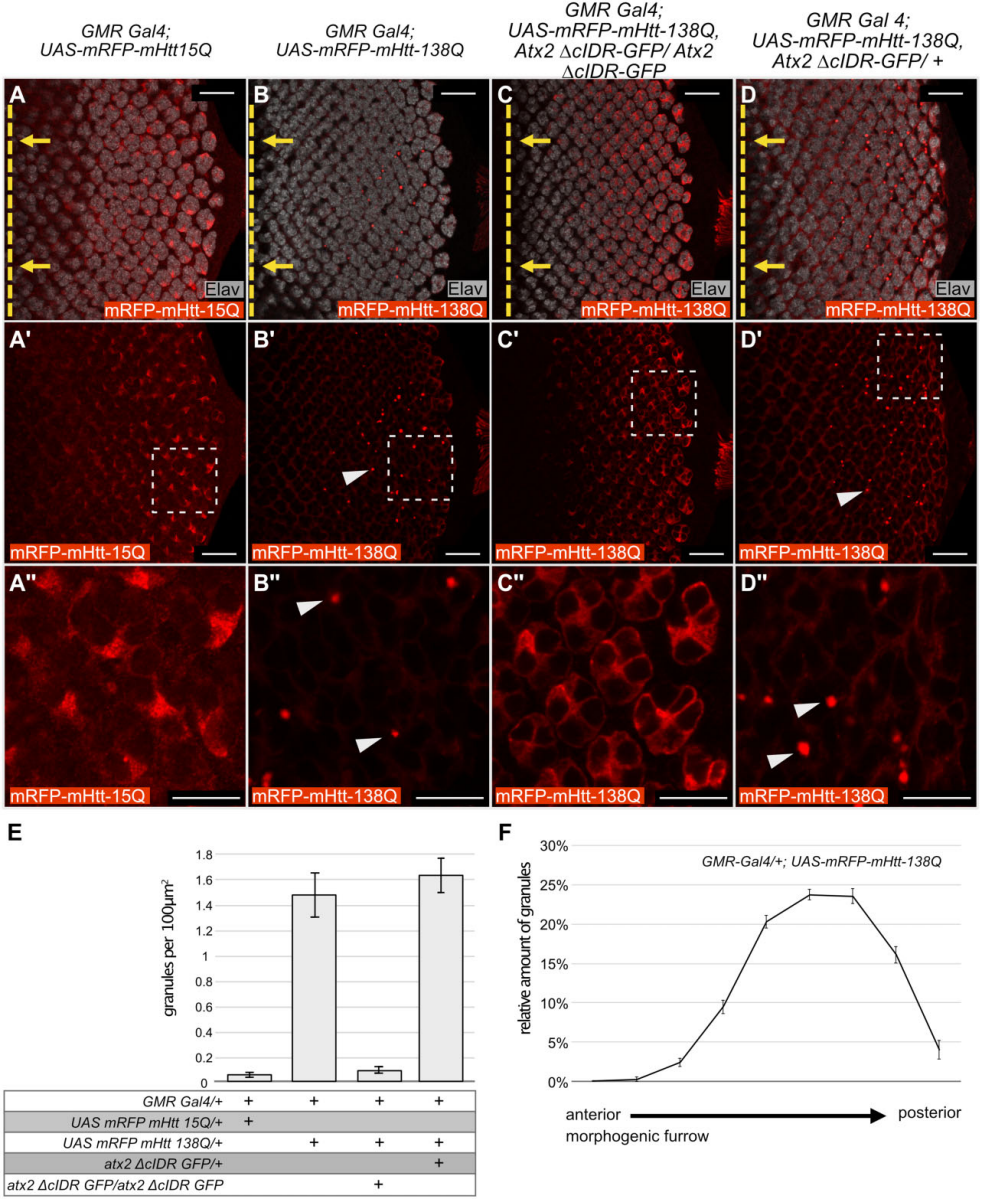
Atx2-cIDR is required for aggregation of Htt-polyQ in *Drosophila* eye discs. (A–D’’) Confocal images, single optical sections, of eye imaginal discs from *Drosophila* third-instar larvae expressing different *UAS-mRFP-mHtt-polyQ* constructs with *GMR-Gal4*. Red: anti-RFP to visualize mHtt-polyQ. Gray: anti-Elav to identify neurons (photoreceptor cells) in the developing disc. Posterior is to the right, the position of the morphogenic furrow is indicated by a dashed yellow line in A–D. Yellow arrows indicate the direction of relative movement of the morphogenic furrow. (A’’–D’’) are 4x magnifications of the indicated areas in A'–D' respectively. No aggregation is observed for *UAS-mRFP-mHtt-15Q* (A–A’’). When over expressed mRFP-mHtt-138Q assembles into aggregates (arrowheads in B’,B’’). This aggregation is dependent on the Atx2 cIDR, as aggregates do not form when the cIDR is not present (C–C’’). The effect of the Atx2 cIDR is not dominant as aggregates of mRFP-mHtt-138Q still form when wild-type Atx2 is present (arrow heads in D’,D’’). Scale bars (A–D’) 20 μm, (A’’–D’’) 10 μm. (E) Quantification of the amount of Huntingtin aggregates in A–D. Bar graphs represent the amounts of aggregates observed per 100 μm^2^. Genotypes are indicated below the graph. From left to right: (1) *GMR Gal4/+; UAS-mRFP-mHtt-15Q/+* (*n* = 8) (2) *GMR Gal4/+; UAS mRFP-mHtt-138Q/+* (*n* = 8) (3) *GMR Gal4/+; UAS mRFP-mHtt-138Q, atx2-ΔcIDR-GFP/atx2-ΔcIDR-GFP* (*n* = 9) (4) *GMR Gal4/+; UAS mRFP-mHtt-138Q/atx2-ΔcIDR-GFP/+* (*n* = 3). Error bars are SEM. (F) Distribution of mRFP-mHtt-138Q aggregates along the anterior-posterior axis of larval eye imaginal discs starting at the morphogenic furrow (left hand side of the graph) to the posterior edge of the imaginal disc (right hand side of the graph) (*n* = 8). Error bars are SEM. The number of aggregates increases with distance to the morphogenic furrow.

For immunofluorescence experiments, mHtt-polyQ granules were identified using the Analyse Particle function in ImageJ. In short, z-projections of confocal images of 3^rd^ instar larvae eye discs were cropped to include the entire GMR-Gal4 expressing domain as defined by the anti-elav staining. Images were blurred (Gaussian blur, Sigma = 2), thresholded and despeckled to create a binary image for particle detection. The Analyse Particle function of imageJ was used to identify aggregates (parameters: size = 50-Infinity pixel circularity = 0.70–1.00). The number of discs per analyzed genotype is listed in Figure 2, the average size of the area analyzed per disc was 15,665 μm^2^.

Atx2 expression levels were calculated based on confocal image stacks collected from eye imaginal discs of either CantonS or *atx2-ΔcIDR-GFP* 3rd instar larvae. To ensure the antibody signal between genotypes was not affected by deviations in the staining protocol between reaction vials, we carried out the staining protocol for both genotypes in the same vials. When scanning we distinguished between the two genotypes based on a dsRed expression only present in the Bolwig nerve of *atx2-ΔcIDR-GFP* flies. We measured the mean fluorescent intensity of Atx2 across multiple levels of the disc using ImageJ. Measurement was limited to the GMR-Gal4 expression domain. The domain was approximated based on the antibody staining for anti-elav. Three independent repeats of the experiment were carried out, with six imaginal discs analyzed per replicate and genotype. For each imaginal disc the relative mean fluorescent intensity in relation to the mean fluorescent intensity of Atx2 signal in the Canton-S control was calculated.

## Results

### Atx2-cIDR is partially required for the progression of mHtt-polyQ induced degeneration

One of the hallmarks of Huntington’s disease is the presence of intracellular aggregates of polyglutamine containing Htt protein fragments. Expression of CAG expanded Htt in the *Drosophila* eye leads to photoreceptor degeneration and disorganization of the ommatidial units of the compound eye (Romero *et al.* 2008). We first confirmed these previous observations and then, to determine if the RNP-granule forming activity of *Drosphila* Atx-2 contributes to Htt-polyQ induced degeneration, tested if degenerative phenotypes were altered if the granule-assembly domain of Atx2 was deleted.

We expressed *UAS-mRFP-mHtt-polyQ* constructs in the eye via the *GMR-Gal4* driver, which induces expression of UAS effectors in all cells posterior to the morphogenetic furrow of the eye imaginal discs (Freeman 1996). Eyes of flies expressing *UAS-mRFP-mHtt-15Q* (a nonpathogenic 15Q repeat) with *GMR-Gal4* were indistinguishable from control flies and did not exhibit any macroscopic degradation (Figure 1, A–B’’’). In contrast, expression of the CAG expanded *UAS-mRFP-mHtt-138Q* construct led to a clearly visible and progressive degeneration of the compound eye with age (Figure 1, C and C’’’). A small subset of flies developed mild localized discoloration of the ommatidia at day 20 after hatching (Figure 1C’’’). By day 50, this discoloration is visible in almost all experimental flies (Figure 1, C’’and C’’’). A similar phenotype can be observed in flies expressing *UAS-Htt-ex1-Q97*. Here the phenotype is already apparent from the first day of hatching (Figure 1, E–E’’’). We then asked how these phenotypes were affected by the removal of the cIDR domain of Atx2, which is required for RNP-granule assembly. Western blotting confirmed that levels of mRFP-mHtt-138Q were similar in experimental *GMR-Gal4/+; UAS-mRFP-mHtt-138Q*, *atx2-ΔcIDR-GFP/atx2-ΔcIDR-GFP* and *GMR-Gal4/+; UAS-mRFP-mHtt-138Q* in *Drosophila* head extracts (Figure 1, J/K, one-tailed Student *t*-test, *P* = 0.306). The specificity of the immunoreactivity was confirmed as no RFP protein is detected in *GMR-Gal4* driver controls. Surprisingly, we detected expression of the *UAS-mRFP-mHtt-138Q* allele in absence of the *GMR-Gal4* driver. Therefore, we anticipated that any phenotypic difference would be due to the change in activity of the Atx2 protein.

To test whether mHtt-polyQ induced degeneration of the eye is dependent on the Atx2-cIDR, we crossed either *UAS-Htt-ex1-Q97* or *UAS-mRFP-mHtt-138Q* into genetic backgrounds where the Atx2-cIDR was deleted via CRISPR mediated genome engineering (Bakthavachalu *et al.* 2018). In both cases, we observed a suppression of the degenerative phenotypes (Figure 1, D–D’’ and F–F’’). In the case of mRFP-mHtt-138Q we could not observe any discoloration in fly eyes even 50 days after eclosion (Figure 1, D–D’’). The difference in phenotype is significant at day 30 and 50 (chi-squared test *P* < 0.00001) but not at day 1 (chi-squared test *P* = 0.265). The phenotype induced by *UAS-Htt-ex1-Q97* in freshly eclosed flies is suppressed by the removal of Atx2-cIDR (Figure 1, E–E’’ and F–F’’) and is significantly different from the phenotype observed in one day old *GMR; UAS-Htt-ex1-Q97* flies (chi-squared test *P* < 0.00001). However, for *UAS-Htt-ex1-Q97* this suppression is no longer evident after 10 days (chi-squared test at 30 days *P* = 0.1134, at 50 days *P* = 0.1241) (Figure 1, F’–F’’).

To address the possibility that suppression of mHtt-polyQ degeneration is due to reduced expression levels of the *atx2-ΔcIDR-GFP* mutant, we compared the levels of Atx2 in eye imaginal discs of homozygous *atx2-ΔcIDR-GFP* and CantonS flies. Homozygous *atx2-ΔcIDR-GFP* flies do not contain any wild-type Atx2, hence immunoreactivity to the Atx2 antibody reports presence of the Atx2-ΔcIDR-GFP protein only (Bakthavachalu *et al.* 2018). We found that in both genotypes Atx2 is similarly distributed in the cells of the eye imaginal disc (Figure 1, G–H’). We noted that the level of Atx2 quantified using immunofluorescence in homozygous *atx2-ΔcIDR-GFP* flies is slightly (∼20%) but significantly lower when compared to the expression level in Canton-S flies (Figure 1I, two-tailed Student’s *t*-Test, *P* = 0.0000069). However, this small reduction, which may indicate a subtle increase in diffuse, less easily measured cytoplasmic labeling, seems unlikely to account for the observed phenotypic differences. As previously shown, the immunoreactivity we observe is specific to the Atx2 protein (Singh *et al.* 2021). Taken together, these observations indicate that as for *Drosophila* ALS models, progressive cytotoxicity in *Drosophila* Huntington’s models is facilitated by the Atx2-cIDR.

An unexpected caveat to this arises from our observation is that the survival rates of flies expressing mHtt-polyQ in *atx2-ΔcIDR-GFP* background are reduced when compared to wild-type controls (Figure 1, inserts in A–F’’, Supplementary Figure S1). Thus, we cannot exclude the possibility that the above experiments inadvertently compare eyes of a healthy subset of experimental flies, for instance with low levels of Htt-polyQ, with a broader range of control flies. While the caveat needs to be noted, we note that prior work on Atx2 has shown that the protein itself is required for Htt-polyQ induced toxicity in clock neurons (Xu *et al.* 2019b). Furthermore, suppression of the phenotype induced by *UAS-Htt-ex1-Q97* in the *atx2-ΔcIDR-GFP* background is observed well before excessive lethality becomes an issue. Thus, our results are indicative of a likely role for the cIDR promoting Htt-polyQ induced toxicity.

To further examine this working hypothesis, we tested whether the formation or stability of Htt-polyQ aggregates observed in Htt-polyQ expressing eye imaginal disc cells were affected by deletion of the cIDR.

### Atx2-cIDR is required for the aggregation of mHtt-polyQ protein

To test whether the Atx2-cIDR is required for aggregation of mHtt-polyQ proteins *in vivo*, we stained and visualized mRFP-mHtt-138Q inclusions in *Drosophila* eye imaginal discs. In control flies expressing the short glutamine-repeat fragment *UAS-mRFP-mHtt-15Q* with *GMR-Gal4*, mHtt-15Q protein appeared diffuse and cytoplasmic in photoreceptor progenitor cells and no inclusions could be seen (Figure 2, A–A’’/E, 0.04 granules per 100 μm^2^). In contrast, in eye-imaginal disc cells expressing the pathogenic polyQ expansion fragment *UAS-mRFP-mHtt-138Q*, we observed obvious cytoplasmic inclusions of the mutant protein (Figure 2, B–B’’/E, 1.01 granules per 100 μm^2^). The presence of mRFP-mHtt-138Q aggregates was predominantly found in the posterior half of the imaginal disc (Figure 2F). This argues for increased aggregation over time, as cells near the optic stalk (posterior) are older as compared to cells close to the morphogenetic furrow (anterior) (Ready *et al.* 1976). This increased aggregation over time is broadly consistent with the late onset of the macroscopically visible phenotype observed in adult *Drosophila* eyes (Figure 1, C–C’’). When expressed in larvae homozygous for the Atx2-cIDR deletion, the mRFP-mHtt-138Q protein distribution is strikingly changed to a diffuse and cytoplasmic pattern, without any apparent aggregates or inclusions (Figure 2, C–C’’/E A, 0.07 granules per 100 μm^2^). Additional control experiments show mHtt-138Q-RFP inclusions in control flies heterozygous for *atx2-ΔcIDR-GFP* (Figure 2, D–D’’/E, 1.29 granules per 100 μm^2^) that still carry one wild-type copy of Atx2. The lack of mHtt-138Q-RFP aggregates in the absence of the Atx2-cIDR argues that Atx2-dependent granules contribute to mRFP-mHtt-138Q aggregate accumulation and indicate a shared mechanism for aggregate formation across at least two different classes of ND. It is interesting that while Htt aggregates can form as early as the third-instar larval stage, degeneration of the adult eye is only obvious around 30 days after eclosion for *mRFP-mHtt-138Q* and 10 days after eclosion for *Htt-ex1-Q97*. Additional experiments are required to understand how Htt aggregates are connected to cell death. One possibility is that aggregates are tolerated in developing imaginal discs, and cause cell death only when their levels or their negative effects on cell metabolism build up over days. Alternatively, it may be that eye defects we score under a dissection microscope do not reveal low levels of cell death that may occur, but be invisible to external inspection, in younger flies.

### mHtt-polyQ aggregates do not sequester stress granule markers

**Figure 3:**
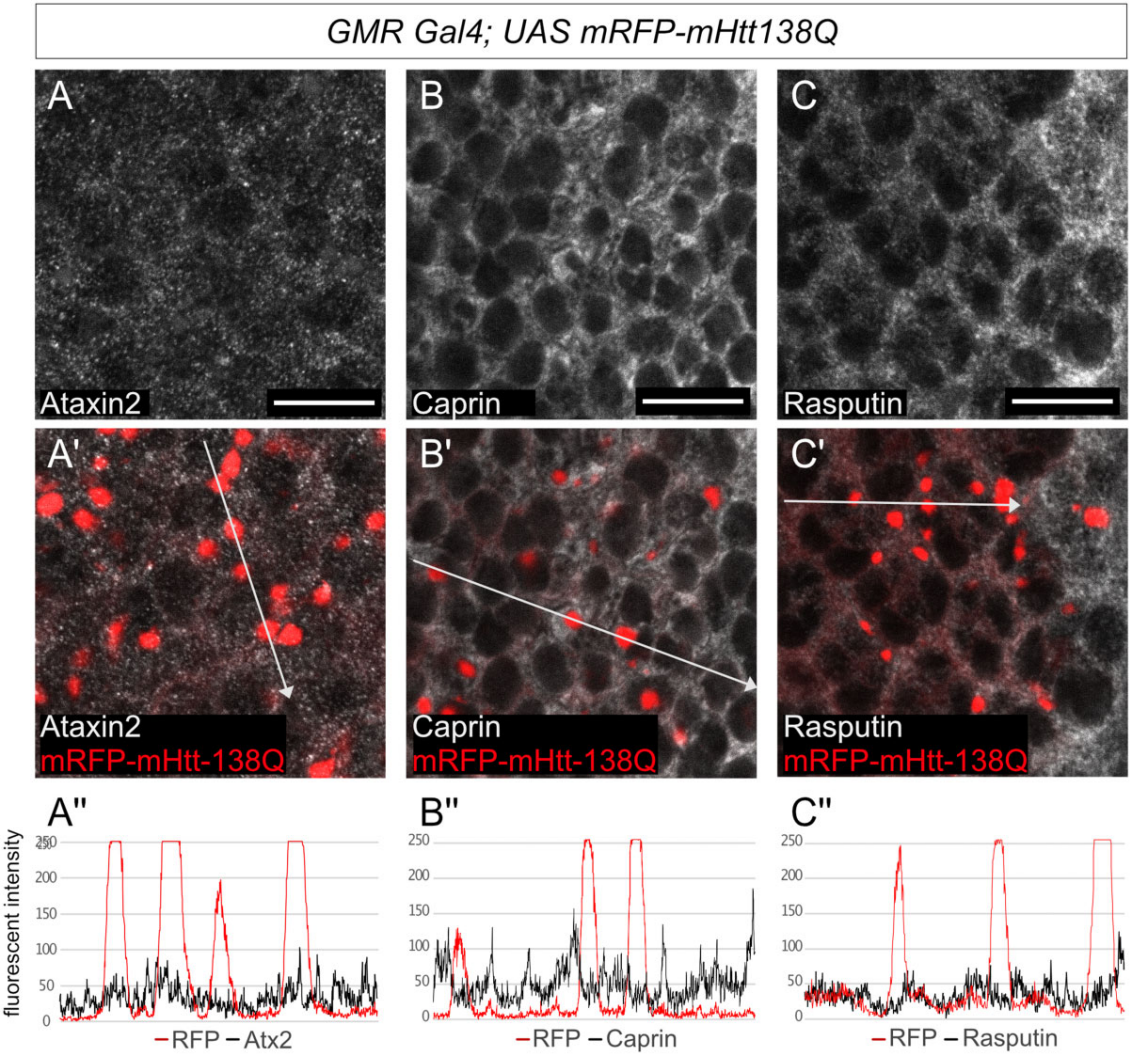
Ectopic polyQ Huntingtin aggregates do not colocalize with stress granule markers. (A–C') Confocal images of *Drosophila* eye discs from third-instar larvae expressing *UAS-mRFP-mHtt-138Q* with *GMR Gal4*. red: mRFP-mHtt-138Q shown by RFP-fluorescence, gray: A–A' anti-Atx2, B-B' anti-Rasputin, in C–C’ anti-Caprin. Posterior is to the right. Scale bars 10μm. (A’’–C’’) Fluorescence intensity plots of the antibody signals measured along the white arrow in A’–C’ respectively. None of Atx2, Caprin nor Rasputin colocalize with mRFP-mHtt-138Q aggregates.

The ALS associated C9-dipeptide repeat and FUS proteins are known to localize substantially to RNA stress granules in cultured cells and/or to inclusions that contain stress granule markers like TDP-43 or Ataxin-2 *in vivo* (Liu-Yesucevitz et al. 2010; Bentmann *et al.* 2012; Bakthavachalu *et al.* 2018; Chew *et al.* 2019) To test whether Htt-polyQ granules also similarly sequester stress-granule markers, we examined Htt-polyQ aggregates *in vivo* and in eye imaginal discs for colocalization with stress granule markers. None of the stress-granule marker proteins tested, including Atx2, Caprin, and Rasputin (G3BP), showed colocalization with mHtt-138QRFP in imaginal disc cells (Figure 3, A–C’’). These experiments do not provide any additional insight into how Atx2 facilitates mHtt-polyQ aggregate formation but also do not, on their own, rule out a transient role for stress granules in early stages of mHtt-138QRFP aggregate formation.

## Discussion

We previously showed that the cIDR of Atx2 is required for the progression of degenerative phenotypes in two ALS models in the fly: C9orf72 di-peptide and FUS (Bakthavachalu *et al.* 2018). In the course of this study, we performed additional experiments to allow us to extend this conclusion to *Drosophila* TDP-43 models for ALS. Overexpression of each *UAS-TDP-43-wt* and *UAS-TDP-43-M337V* in the fly eye leads to degeneration of the ommatidia with different degrees of severity (Ritson *et al.* 2010). The *TDP-43-M337V* mutation, which results in the substitution of residues in a phylogenetically conserved region of TDP-43, is linked to familial cases of ALS (Sreedharan *et al.* 2008). While previous experiments have demonstrated that TDP-43 induced cytotoxicity is dependent on Atx2 (Ritson *et al.* 2010) our current analysis additionally shows that degeneration of the fly eye in TDP-43 models is facilitated by the Atx2-cIDR (Supplementary Figure S2 and legend).

Given that the proteins encoded by several ALS genes associate with stress granules, a role for RNP granules in promoting the formation of ALS-associated inclusions containing RNP granule components can be logically rationalized (Bakthavachalu *et al.* 2018; Becker and Gitler 2018). But could Ataxin-2 and its role in RNP-granule assembly also be relevant to other forms of neurodegeneration? Previous research in *Drosophila* has demonstrated the role of Ataxin-2 in the progression of multiple poly-glutamine-repeat diseases. Modulation of Ataxin-2 levels affects the severity of neurodegenerative phenotypes in *Drosophila* models of SCA1 and SCA3. In both disease models reduction of Ataxin-2 levels act neuroprotective whereas increased Ataxin-2 levels enhance the degenerative phenotypes (Al-Ramahi *et al*. 2007; Lessing and Bonini 2008). Ectopic expression of human Htt-polyQ protein has previously been shown to induce granules in the cytoplasm of larval photoreceptor cells. Interestingly in adult flies Htt-polyQ protein predominantly re-locates to the nucleus of photoreceptor neurons with increased age (Jackson *et al.* 1998). A recent study on Huntington’s Disease showed that overexpression of different mutant polyQ Huntington transgenes in circadian clock neurons induces circadian arrhythmicity and aggregation of Htt-polyQ in small ventral lateral neurons (sLNVs) as well as reduction in cell numbers indicative of cytotoxicity (Xu *et al.* 2019a, 2019b). Reduction of Atx2 via RNAi knockdown alleviates both of these effects (Xu *et al.* 2019b). As removal of the Atx2-cIDR has a similar effect in larval photoreceptor cells as the knock down of Atx2 in adult clock neurons, it is likely, that the formation of further aggregates and their subsequent re-localization to the nucleus in adult flies is also inhibited. However, the precise activities of Atx2 required to promote degeneration or inclusion formation in clock neurons remains unclear.

Here, we report the novel observation that the Atx2-cIDR is required for progression of neurodegenerative phenotypes as well as protein inclusion formation in fly Huntington’s disease models. This observation, which suggests a role for Atx2-dependent macromolecular assembles in HD progression, is difficult to explain because not only is Htt not obviously a component of known Atx2 containing RNP granules, but Htt-polyQ inclusions are also not known to contain additional RNP granule components. Thus, these data point to less obvious cell biological mechanisms that connect Atx2 mediated RNP granules to Htt-polyQ proteinopathy. We suggest three broad mechanisms to explain these observations.

### Model 1: Huntingtin assembles transiently into an undefined Atx2-dependent macromolecular complex

It is possible, that the granule-forming properties of Atx2-cIDR are not limited to stress granules, but can be extended to other phase separating complexes as well. There are reports that upon heat stress, endogenous Htt protein rapidly forms granules, termed Huntington Stress Bodies (HSB). These granules are reversible and may not colocalize with classical processing body and stress granule markers after heat shock (Nath *et al.* 2015). Thus, there is a possibility that a new class of RNP granule or non-RNP complex exists in which Htt can be concentrated, whose assembly may be dependent on Atx2. Loss of the Atx2-cIDR may prevent assembly of this complex, and by eliminating a structure in which Htt-polyQ is concentrated, thereby reduce opportunities for the protein to aggregate. In this context, an interesting recent study in *Drosophila* concludes that both the expanded poly-Q domain and flanking sequences impact on cellular distribution and pathology of mutant Htt, suggesting that a specific type of aggregate, and not-aggregation alone, drive neurodegeneration in HD (Chongtham *et al.* 2020).

### Model 2: Misfolded Huntingtin is dynamically concentrated in stress granules

It is possible that misfolded proteins with no function in translational control are also transiently concentrated and can assemble within stress granules. Consistent with this, recent studies in mammalian cells have shown that stress granules may serve an additional function in protein handling (Ganassi *et al.* 2016; Mateju *et al.* 2017). Time-lapse microscopy has demonstrated that a pathogenic, misfolded form of superoxide dismutase (SOD) can associate with a subset of stress granules, which first become less dynamic and then gradually evolve into pure SOD inclusions (Mateju *et al.* 2017). Defective Ribosomal Products (DRiPs) also accumulate in stress granules and promote an aberrant state, which is resistant to RNAses (Ganassi *et al.* 2016). These observations can be rationalized as follows. Granules being held together by disordered domain interactions, as facilitated by Atx2, may provide an environment in which proteins that are misfolded under conditions of stress can be stabilized, perhaps refolded, and returned to the cytoplasm. Thus, SGs have a role in normal physiological protein handling (Alberti *et al.* 2017; Alberti and Hyman 2021). However, pathogenic and assembly-prone forms of misfolded proteins when concentrated in SGs may efficiently form amyloid-rich aggregates that exclude native SG proteins. In this scenario, Atx2 cIDR deletion would inhibit SG assembly and reduce inclusion body formation by eliminating the microenvironment in which such pathogenic proteins are transiently concentrated. Although we do not observe the proposed transient colocalization of Htt-polyQ with SG markers in our experiments, there are previous observations consistent with such a model. In particular, after ER stress, Htt-polyQ has been reported to colocalize with Caprin1 and G3BP1 in cultured cells (Ratovitski *et al.* 2012). In a cultured Drosophila S2 cells dsRNA screen, knock down of Rasputin, the *Drosophila* homolog of G3BP1/2, has been shown to reduce Htt-polyQ aggregation (Zhang *et al.* 2010).

### Model 3: Absence of the Atx2 cIDR increases the pool of available chaperones or neuroprotective proteins

Physiological stress granules contain multiple chaperones, at least some of which have been shown to regulate their assembly and disassembly (Ganassi *et al.* 2016; Jain *et al.* 2016). A potential role for these chaperones may be to prevent stable aggregation of SG components when they are highly concentrated within RNP granules. It is therefore possible that if RNP granules are present at much lower levels in cells lacking the Atx2-cIDR, then higher levels of free soluble chaperones could be available for handling unrelated protein aggregates, such as those formed by polyQ-expanded forms of Htt.

Taken together, our experiments indicate an unexpected role for the granule forming ability of Atx2 in the progression of Huntington’s disease-related phenotypes in a *Drosophila* model. This points to the possibility that Atx2-targeting therapeutics, which are now in development for SCA2 and ALS, may have wider use for other neurodegenerative conditions. While we propose models for how Atx2-dependent assemblies may have such roles in Huntington’s disease or other proteinopathies, additional experiments will be required to test and establish these or alternative proposals to explain the phenomena reported here.

## Data availability

Strains are available upon request. The data underlying this article are available in the article and in its online supplementary material.

Supplementary material is available at *G3* online.

## Supplementary Material

jkab355_Supplementary_Figure_1

jkab355_Supplementary_Figure_2

## References

[jkab355-B1] Aguilera-Gomez A , Zacharogianni M, van Oorschot MM, Genau H, Grond R, et al 2017. Phospho-rasputin stabilization by sec16 is required for stress granule formation upon amino acid starvation. Cell Rep. 20:935–948. 28746877 10.1016/j.celrep.2017.06.042 PMC6064189

[jkab355-B2] Alberti S , Hyman AA. 2021. Biomolecular condensates at the nexus of cellular stress, protein aggregation disease and ageing. Nat Rev Mol Cell Biol. 22:196–213. 33510441 10.1038/s41580-020-00326-6

[jkab355-B3] Alberti S , Mateju D, Mediani L, Carra S. 2017. Granulostasis: protein quality control of RNP granules. Front Mol Neurosci. 10:84. 28396624 10.3389/fnmol.2017.00084 PMC5367262

[jkab355-B4] Al-Ramahi I , Pérez AM, Lim J, Zhang M, Sorensen R, et al 2007a. dAtaxin-2 mediates expanded ataxin-1-induced neurodegeneration in a Drosophila model of SCA1. PLoS Genet. 3:e234. 18166084 10.1371/journal.pgen.0030234 PMC2323314

[jkab355-B5] Bakthavachalu B , Huelsmeier J, Sudhakaran IP, Hillebrand J, Singh A, et al 2018. RNP-granule assembly via Ataxin-2 disordered domains is required for long-term memory and neurodegeneration. Neuron. 98:754–766.e4. 29772202 10.1016/j.neuron.2018.04.032

[jkab355-B6] Becker LA , Gitler AD. 2018. Ataxin-2 is Droppin’ some knowledge. Neuron. 98:673–675. 29772196 10.1016/j.neuron.2018.05.002

[jkab355-B7] Becker LA , Huang B, Bieri G, Ma R, Knowles DA, et al 2017. Therapeutic reduction of ataxin-2 extends lifespan and reduces pathology in TDP-43 mice. Nature. 544:367–371. 28405022 10.1038/nature22038 PMC5642042

[jkab355-B8] Bentmann E , Neumann M, Tahirovic S, Rodde R, Dormann D, et al 2012. Requirements for stress granule recruitment of fused in sarcoma (FUS) and TAR DNA-binding protein of 43 kDa (TDP-43). J Biol Chem. 287:23079–23094. 22563080 10.1074/jbc.M111.328757 PMC3391091

[jkab355-B9] Braak H , Brettschneider J, Ludolph AC, Lee VM, Trojanowski JQ, et al 2013. Amyotrophic lateral sclerosis—a model of corticofugal axonal spread. Nat Rev Neurol. 9:708–714. 24217521 10.1038/nrneurol.2013.221 PMC3943211

[jkab355-B10] Chew J , Cook C, Gendron TF, Jansen-West K, Rosso GD, et al 2019. Aberrant deposition of stress granule-resident proteins linked to C9orf72-associated TDP-43 proteinopathy. Mol Neurodegener. 14:9–15. 30767771 10.1186/s13024-019-0310-z PMC6377782

[jkab355-B11] Chongtham A , Bornemann DJ, Barbaro BA, Lukacsovich T, Agrawal N, et al 2020. Effects of flanking sequences and cellular context on subcellular behavior and pathology of mutant HTT. Hum Mol Genet. 29:674–688. 31943010 10.1093/hmg/ddaa001 PMC7608755

[jkab355-B12] Duennwald ML , Lindquist S. 2008. Impaired ERAD and ER stress are early and specific events in polyglutamine toxicity. Genes Dev. 22:3308–3319. 19015277 10.1101/gad.1673408 PMC2600758

[jkab355-B13] Duyckaerts C , Delatour B, Potier MC. 2009. Classification and basic pathology of Alzheimer's disease. Acta Neuropathol. 118:5–36. 19381658 10.1007/s00401-009-0532-1

[jkab355-B14] Elden AC , Kim H-J, Hart MP, Chen-Plotkin AS, Johnson BS, et al 2010. Ataxin-2 intermediate-length polyglutamine expansions are associated with increased risk for ALS. Nature. 466:1069–1075. 20740007 10.1038/nature09320 PMC2965417

[jkab355-B15] Freeman M. 1996. Reiterative use of the EGF receptor triggers differentiation of all cell types in the Drosophila eye. Cell. 87:651–660. 8929534 10.1016/s0092-8674(00)81385-9

[jkab355-B16] Freibaum BD , Lu Y, Lopez-Gonzalez R, Kim NC, Almeida S, et al 2015. GGGGCC repeat expansion in C9orf72 compromises nucleocytoplasmic transport. Nature. 525:129–133. 26308899 10.1038/nature14974 PMC4631399

[jkab355-B17] Ganassi M , Mateju D, Bigi I, Mediani L, Poser I, et al 2016. A surveillance function of the HSPB8-BAG3-HSP70 chaperone complex ensures stress granule integrity and dynamism. Mol Cell. 63:796–810. 27570075 10.1016/j.molcel.2016.07.021

[jkab355-B18] Ganz J , Shacham T, Kramer M, Shenkman M, Eiger H, et al 2020. A novel specific PERK activator reduces toxicity and extends survival in Huntington’s disease models. Sci Rep. 10:1–15. 32327686 10.1038/s41598-020-63899-4 PMC7181660

[jkab355-B19] Gopal PP , Nirschl JJ, Klinman E, Holzbaur ELF. 2017. Amyotrophic lateral sclerosis-linked mutations increase the viscosity of liquid-like TDP-43 RNP granules in neurons. Proc Natl Acad Sci USA. 114:E2466–E2475. 28265061 10.1073/pnas.1614462114 PMC5373408

[jkab355-B20] Grima JC , Daigle JG, Arbez N, Cunningham KC, Zhang K, et al 2017. Mutant huntingtin disrupts the nuclear pore complex. Neuron. 94:93–107.e6. 28384479 10.1016/j.neuron.2017.03.023 PMC5595097

[jkab355-B21] Guo W , Chen Y, Zhou X, Kar A, Ray P, et al 2011. An ALS-associated mutation affecting TDP-43 enhances protein aggregation, fibril formation and neurotoxicity. Nat Struct Mol Biol. 18:822–830. 21666678 10.1038/nsmb.2053 PMC3357956

[jkab355-B22] Halliday M , Radford H, Sekine Y, Moreno J, Verity N, et al 2015. Partial restoration of protein synthesis rates by the small molecule ISRIB prevents neurodegeneration without pancreatic toxicity. Cell Death Dis. 6:e1672. 25741597 10.1038/cddis.2015.49 PMC4385927

[jkab355-B23] Halliday M , Radford H, Zents KAM, Molloy C, Moreno JA, et al 2017. Repurposed drugs targeting eIF2α-P-mediated translational repression prevent neurodegeneration in mice. Brain. 140:1768–1783. 28430857 10.1093/brain/awx074 PMC5445255

[jkab355-B24] Harding HP , Zhang Y, Zeng H, Novoa I, Lu PD, et al 2003. An integrated stress response regulates amino acid metabolism and resistance to oxidative stress. Mol Cell. 11:619–633. 12667446 10.1016/s1097-2765(03)00105-9

[jkab355-B25] Hart MP , Brettschneider J, Lee VMY, Trojanowski JQ, Gitler AD. 2012. Distinct TDP-43 pathology in ALS patients with ataxin 2 intermediate-length polyQ expansions. Acta Neuropathol. 124:221–230. 22526021 10.1007/s00401-012-0985-5 PMC3543833

[jkab355-B26] Henderson MX , Trojanowski JQ, Lee VMY. 2019. α-Synuclein pathology in Parkinson’s disease and related α-synucleinopathies. Neurosci Lett. 709:134316. 31170426 10.1016/j.neulet.2019.134316 PMC7014913

[jkab355-B27] Huynh DP , Figueroa K, Hoang N, Pulst SM. 2000. Nuclear localization or inclusion body formation of ataxin-2 are not necessary for SCA2 pathogenesis in mouse or human. Nat Genet. 26:44–50. 10973246 10.1038/79162

[jkab355-B28] Inagaki H , Hosoda N, Tsuiji H, Hoshino SI. 2020. Direct evidence that ataxin-2 is a translational activator mediating cytoplasmic polyadenylation. J Biol Chem. 295:15810–15825. 32989052 10.1074/jbc.RA120.013835 PMC7681009

[jkab355-B29] Jackson GR , Salecker I, Dong X, Yao X, Arnheim N, et al 1998. Polyglutamine-expanded human huntingtin transgenes induce degeneration of Drosophila photoreceptor neurons. Neuron. 21:633–642. 9768849 10.1016/s0896-6273(00)80573-5

[jkab355-B30] Jain S , Wheeler JR, Walters RW, Agrawal A, Barsic A, et al 2016. ATPase-modulated stress granules contain a diverse proteome and substructure. Cell. 164:487–498. 26777405 10.1016/j.cell.2015.12.038 PMC4733397

[jkab355-B31] Jonsson T , Atwal JK, Steinberg S, Snaedal J, Jonsson PV, et al 2012. A mutation in APP protects against Alzheimer’s disease and age-related cognitive decline. Nature. 488:96–99. 22801501 10.1038/nature11283

[jkab355-B32] Kim HJ , Kim NC, Wang Y-D, Scarborough EA, Moore J, et al 2013. Mutations in prion-like domains in hnRNPA2B1 and hnRNPA1 cause multisystem proteinopathy and ALS. Nature. 495:467–473. 23455423 10.1038/nature11922 PMC3756911

[jkab355-B33] Landles C , Sathasivam K, Weiss A, Woodman B, Moffitt H, et al 2010. Proteolysis of mutant huntingtin produces an exon 1 fragment that accumulates as an aggregated protein in neuronal nuclei in Huntington disease. J Biol Chem. 285:8808–8823. 20086007 10.1074/jbc.M109.075028 PMC2838303

[jkab355-B34] Lastres-Becker I , Rüb U, Auburger G. 2008. Spinocerebellar ataxia 2 (SCA2). Cerebellum. 7:115–124. 18418684 10.1007/s12311-008-0019-y

[jkab355-B35] Lessing D , Bonini NM. 2008. Polyglutamine genes interact to modulate the severity and progression of neurodegeneration in Drosophila. PLoS Biol. 6:e29. 18271626 10.1371/journal.pbio.0060029 PMC2235903

[jkab355-B36] Lim C , Allada R. 2013. ATAXIN-2 activates PERIOD translation to sustain circadian rhythms in Drosophila. Science. 340:875–879. 23687047 10.1126/science.1234785

[jkab355-B37] Liu-Yesucevitz L , Bilgutay A, Zhang Y-J, Vanderwyde T, Citro A, et al 2010. Tar DNA binding protein-43 (TDP-43) associates with stress granules: analysis of cultured cells and pathological brain tissue. PLoS One. 5:e13250. 20948999 10.1371/journal.pone.0013250 PMC2952586

[jkab355-B38] Luk KC , Kehm V, Carroll J, Zhang B, O'Brien P, et al 2012. Pathological α-synuclein transmission initiates Parkinson-like neurodegeneration in nontransgenic mice. Science. 338:949–953. 23161999 10.1126/science.1227157 PMC3552321

[jkab355-B39] Markmiller S , Soltanieh S, Server KL, Mak R, Jin W, et al 2018. Context-dependent and disease-specific diversity in protein interactions within stress granules. Cell. 172:590–604.e13. 29373831 10.1016/j.cell.2017.12.032 PMC5969999

[jkab355-B40] Mateju D , Franzmann TM, Patel A, Kopach A, Boczek EE, et al 2017. An aberrant phase transition of stress granules triggered by misfolded protein and prevented by chaperone function. EMBO J. 36:1669–1687. 28377462 10.15252/embj.201695957 PMC5470046

[jkab355-B41] McCann C , Holohan EE, Das S, Dervan A, Larkin A, et al 2011. The Ataxin-2 protein is required for microRNA function and synapse-specific long-term olfactory habituation. Proc Natl Acad Sci USA. 108:E655–E662. 21795609 10.1073/pnas.1107198108 PMC3169144

[jkab355-B42] Moreno JA , Radford H, Peretti D, Steinert JR, Verity N, et al 2012. Sustained translational repression by eIF2a-P mediates prion neurodegeneration. Nature. 485:507–511. 22622579 10.1038/nature11058 PMC3378208

[jkab355-B43] Nath S , Munsie LN, Truant R. 2015. A huntingtin-mediated fast stress response halting endosomal trafficking is defective in Huntington’s disease. Hum Mol Genet. 24:450–462. 25205111 10.1093/hmg/ddu460 PMC4275073

[jkab355-B44] Nonhoff U , Ralser M, Welzel F, Piccini I, Balzereit D, et al 2007. Ataxin-2 interacts with the DEAD/H-box RNA helicase DDX6 and interferes with P-bodies and stress granules. Mol Biol Cell. 18:1385–1396. 17392519 10.1091/mbc.E06-12-1120 PMC1838996

[jkab355-B45] Papoulas O , Monzo KF, Cantin GT, Ruse C, Yates JR, et al 2010. dFMRP and Caprin, translational regulators of synaptic plasticity, control the cell cycle at the Drosophila mid-blastula transition. Development. 137:4201–4209. 21068064 10.1242/dev.055046 PMC2990211

[jkab355-B46] Pavitt GD. 2018. Regulation of translation initiation factor eIF2B at the hub of the integrated stress response. Wiley Interdiscip Rev RNA. 9:e1491. 29989343 10.1002/wrna.1491

[jkab355-B47] Peng C , Trojanowski JQ, Lee VMY. 2020. Protein transmission in neurodegenerative disease. Nat Rev Neurol. 16:199–212. 32203399 10.1038/s41582-020-0333-7 PMC9242841

[jkab355-B48] Peskett TR , Rau F, O'Driscoll J, Patani R, Lowe AR, et al 2018. A liquid to solid phase transition underlying pathological Huntingtin Exon1 aggregation. Mol Cell. 70:588–601.e6. 29754822 10.1016/j.molcel.2018.04.007 PMC5971205

[jkab355-B49] Polymeropoulos MH , Lavedan C, Leroy E, Ide SE, Dehejia A, et al 1997. Mutation in the α-synuclein gene identified in families with Parkinson’s disease. Science. 276:2045–2047. 9197268 10.1126/science.276.5321.2045

[jkab355-B50] Prusiner SB. 1991. Molecular biology of prion diseases. Science. 252:1515–1522. 1675487 10.1126/science.1675487

[jkab355-B51] Ramaswami M , Taylor JP, Parker R. 2013. Altered Ribostasis: RNA-protein granules in degenerative disorders. Cell. 154:727–736. 23953108 10.1016/j.cell.2013.07.038 PMC3811119

[jkab355-B52] Ratovitski T , Chighladze E, Arbez N, Boronina T, Herbrich S, et al 2012. Huntingtin protein interactions altered by polyglutamine expansion as determined by quantitative proteomic analysis. Cell Cycle. 11:2006–2021. 22580459 10.4161/cc.20423 PMC3359124

[jkab355-B53] Ready DF , Hanson TE, Benzer S. 1976. Development of the Drosophila retina, a neurocrystalline lattice. Dev Biol. 53:217–240. 825400 10.1016/0012-1606(76)90225-6

[jkab355-B54] Ritson GP , Custer SK, Freibaum BD, Guinto JB, Geffel D, et al 2010. TDP-43 mediates degeneration in a novel Drosophila model of disease caused by mutations in VCP/p97. J Neurosci. 30:7729–7739. 20519548 10.1523/JNEUROSCI.5894-09.2010 PMC2890254

[jkab355-B55] Romero E , Cha G-H, Verstreken P, Ly CV, Hughes RE, et al 2008. Suppression of neurodegeneration and increased neurotransmission caused by expanded full-length Huntingtin accumulating in the cytoplasm. Neuron. 57:27–40. 18184562 10.1016/j.neuron.2007.11.025 PMC2277511

[jkab355-B56] Ross CA , Poirier MA. 2004. Protein aggregation and neurodegenerative disease. Nat Med. 10:S10–S17. 15272267 10.1038/nm1066

[jkab355-B57] Rüb U , Schöls L, Paulson H, Auburger G, Kermer P, et al 2013. Clinical features, neurogenetics and neuropathology of the polyglutamine spinocerebellar ataxias type 1, 2, 3, 6 and 7. Prog Neurobiol. 104:38–66. 23438480 10.1016/j.pneurobio.2013.01.001

[jkab355-B58] Sathasivam K , Neueder A, Gipson TA, Landles C, Benjamin AC, et al 2013. Aberrant splicing of HTT generates the pathogenic exon 1 protein in Huntington disease. Proc Natl Acad Sci USA. 110:2366–2370. 23341618 10.1073/pnas.1221891110 PMC3568346

[jkab355-B59] Schilling J , Broemer M, Atanassov I, Duernberger Y, Vorberg I, et al 2019. Deregulated splicing is a major mechanism of RNA-induced toxicity in Huntington’s disease. J Mol Biol. 431:1869–1877. 30711541 10.1016/j.jmb.2019.01.034

[jkab355-B60] Schneider CA , Rasband WS, Eliceiri KW. 2012. NIH Image to ImageJ: 25 years of image analysis. Nat Methods. 9:671–675. 22930834 10.1038/nmeth.2089 PMC5554542

[jkab355-B61] Scoles DR , Meera P, Schneider MD, Paul S, Dansithong W, et al 2017. Antisense oligonucleotide therapy for spinocerebellar ataxia type 2. Nature. 544:362–366. 28405024 10.1038/nature22044 PMC6625650

[jkab355-B62] Seidel K , Siswanto S, Brunt ERP, Den Dunnen W, Korf HW, et al 2012. Brain pathology of spinocerebellar ataxias. Acta Neuropathol. 124:1–21. 22684686 10.1007/s00401-012-1000-x

[jkab355-B63] Seidel K , Siswanto S, Fredrich M, Bouzrou M, den Dunnen WFA, et al 2017. On the distribution of intranuclear and cytoplasmic aggregates in the brainstem of patients with spinocerebellar ataxia type 2 and 3. Brain Pathol. 27:345–355. 27377427 10.1111/bpa.12412 PMC8028910

[jkab355-B64] Shacham T , Sharma N, Lederkremer GZ. 2019. Protein misfolding and ER stress in Huntington’s disease. Front Mol Biosci. 6:20. 31001537 10.3389/fmolb.2019.00020 PMC6456712

[jkab355-B65] Sidrauski C , McGeachy AM, Ingolia NT, Walter P. 2015. The small molecule ISRIB reverses the effects of eIF2α phosphorylation on translation and stress granule assembly. Elife. 4:e05033. 10.7554/eLife.05033 PMC4341466 25719440

[jkab355-B66] Singh A , Hulsmeier J, Kandi AR, Pothapragada SS, Hillebrand J, et al 2021. Antagonistic roles for ataxin-2 structured and disordered domains in RNP condensation. Elife. 10:e60326. 33689682 10.7554/eLife.60326 PMC7946432

[jkab355-B67] Soto C , Pritzkow S. 2018. Protein misfolding, aggregation, and conformational strains in neurodegenerative diseases. Nat Neurosci. 21:1332–1340. 30250260 10.1038/s41593-018-0235-9 PMC6432913

[jkab355-B68] Spillantini MG , Schmidt ML, Lee VMY, Trojanowski JQ, Jakes R, et al 1997. Alpha-synuclein in Lewy bodies. Nature. 388:839–840. 9278044 10.1038/42166

[jkab355-B69] Sreedharan J , Blair IP, Tripathi VB, Hu X, Vance C, et al 2008. TDP-43 mutations in familial and sporadic amyotrophic lateral sclerosis. Science. 319:1668–1672. 18309045 10.1126/science.1154584 PMC7116650

[jkab355-B70] Sudhakaran IP , Hillebrand J, Dervan A, Das S, Holohan EE, et al 2014. FMRP and Ataxin-2 function together in long-term olfactory habituation and neuronal translational control. Proc Natl Acad Sci USA. 111:E99–E108. 24344294 10.1073/pnas.1309543111 PMC3890871

[jkab355-B71] Taylor JP , Brown RH, Cleveland DW. 2016. Decoding ALS: from genes to mechanism. Nature. 539:197–206. 27830784 10.1038/nature20413 PMC5585017

[jkab355-B72] Vonsattel JP , Myers RH, Stevens TJ, Ferrante RJ, Bird ED, et al 1985. Neuropathological classification of Huntington’s disease. J Neuropathol Exp Neurol. 44:559–577. 2932539 10.1097/00005072-198511000-00003

[jkab355-B73] Weiss KR , Kimura Y, Lee W-CM, Littleton JT. 2012. Huntingtin aggregation kinetics and their pathological role in a Drosophila Huntington’s disease model. Genetics. 190:581–600. 22095086 10.1534/genetics.111.133710 PMC3276644

[jkab355-B74] Wek RC , Jiang H-Y, Anthony TG. 2006. Coping with stress: eIF2 kinases and translational control. Biochem Soc Trans. 34:7–11. 16246168 10.1042/BST20060007

[jkab355-B75] Xu F , Kula-Eversole E, Iwanaszko M, Hutchison AL, Dinner A, et al 2019a. Circadian clocks function in concert with heat shock organizing protein to modulate mutant Huntingtin aggregation and toxicity. Cell Rep. 27:59–70.e4. 30943415 10.1016/j.celrep.2019.03.015 PMC7237104

[jkab355-B76] Xu F , Kula-Eversole E, Iwanaszko M, Lim C, Allada R. 2019b. Ataxin2 functions via CrebA to mediate Huntingtin toxicity in circadian clock neurons. PLoS Genet. 15:e1008356. 31593562 10.1371/journal.pgen.1008356 PMC6782096

[jkab355-B77] Zhang S , Binari R, Zhou R, Perrimon N. 2010. A genomewide RNA interference screen for modifiers of aggregates formation by mutant huntingtin in drosophila. Genetics. 184:1165–1179. 20100940 10.1534/genetics.109.112516 PMC2865916

[jkab355-B78] Zhang K , Donnelly CJ, Haeusler AR, Grima JC, Machamer JB, et al 2015. The C9orf72 repeat expansion disrupts nucleocytoplasmic transport. Nature. 525:56–61. 26308891 10.1038/nature14973 PMC4800742

[jkab355-B79] Zhang Y , Ling J, Yuan C, Dubruille R, Emery P. 2013. A role for Drosophila ATX2 in activation of PER translation and circadian behavior. Science. 340:879–882. 23687048 10.1126/science.1234746 PMC4078874

