# Supplementary material for: A C-terminal ataxin-2 disordered region promotes Huntingtin protein aggregation and neurodegeneration in Drosophila models of Huntington’s disease
Source: G3 (Bethesda). 2021 Oct 9;11(12):jkab355. doi: 10.1093/g3journal/jkab355 (PMC8664476; doi:10.1093/g3journal/jkab355)
Supplement: jkab355_Supplementary_Figure_1 [file jkab355_supplementary_figure_1.pdf]

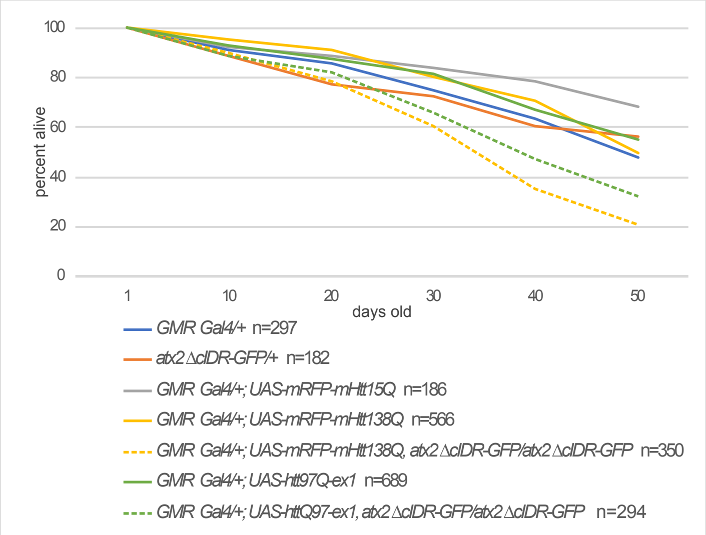

Supplementary Figure 1: Life span of flies expressing pathogenic Htt-polyQ transgenes

Survival rate of flies expressing Htt-polyQ transgenes. Number of flies per genotype at day 1 is listed in the figure legend.
