# Supplementary material for: A C-terminal ataxin-2 disordered region promotes Huntingtin protein aggregation and neurodegeneration in Drosophila models of Huntington’s disease
Source: G3 (Bethesda). 2021 Oct 9;11(12):jkab355. doi: 10.1093/g3journal/jkab355 (PMC8664476; doi:10.1093/g3journal/jkab355)
Supplement: jkab355_Supplementary_Figure_2 [file jkab355_supplementary_figure_2.pdf]

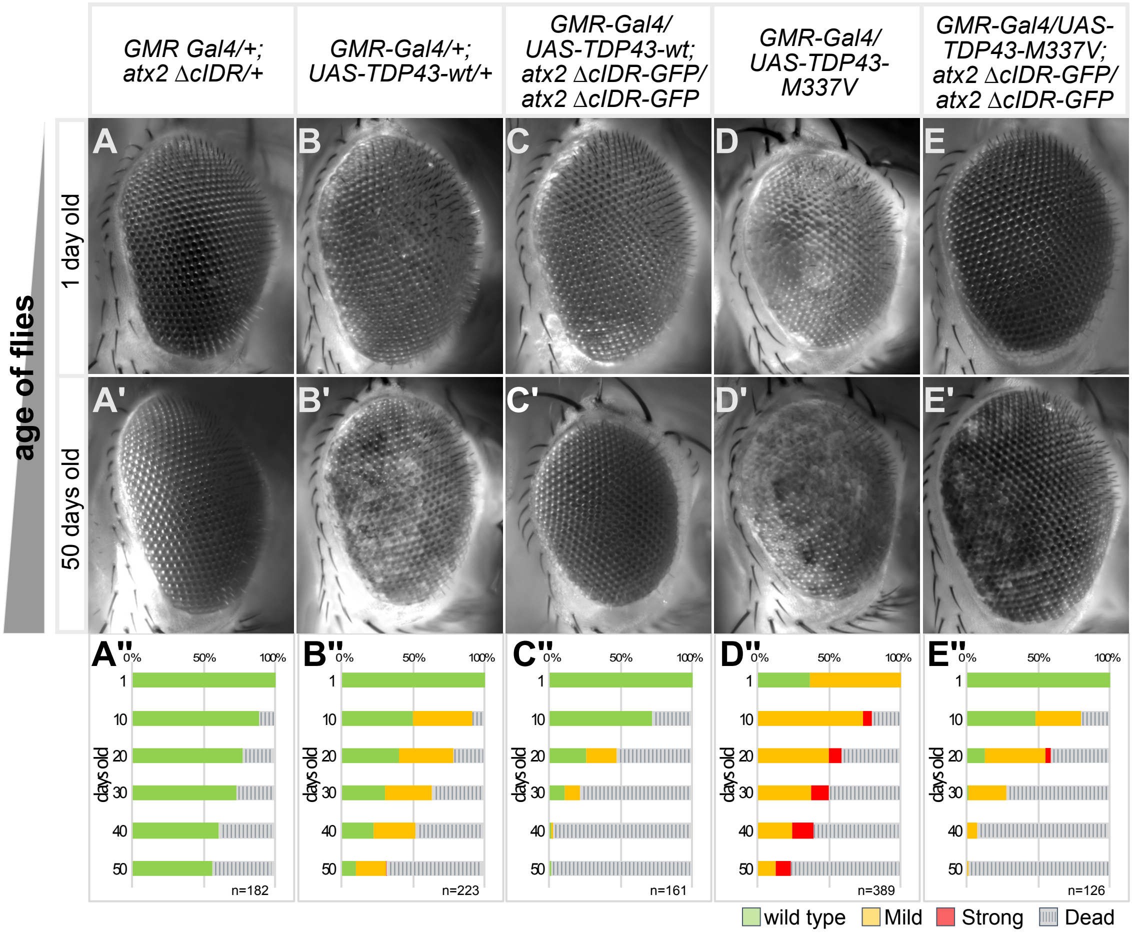

Supplementary Figure 2: Atx2-cIDR is required for TDP43 induced Neurodegeneration.

A-E': Representative images of *Drosophila* eyes taken at the indicated time points (1 day old and 50 days old). Genotypes are indicated at the top of each column. A''-F'' quantification of phenotypes observed at 10-day intervals between 1-day and 50-days after eclosion. Green bars indicate wild-type eyes, yellow bars a mild phenotype with patches of discoloration or rough surface visible, red bars are eyes with necrotic spots, grey bars represent dead flies. The bars are percentages of the flies counted on each day. We note that flies carrying the cIDR deletion appear to have shorter life spans compared to that of TDP43 expressing flies (analogous to what is seen Htt-polyQ expressing animals). This observation could indicate reduced fitness of flies carrying the c-IDR deletion. n per genotype is indicated at bottom right of each bar graph. Significant differences between *GMR-Gal4/+;UAS-TDP43-wt/+* and *GMR-Gal4/UAS-TDP43-wt; atx2 ΔcIDR-GFP/ atx2 ΔcIDR-GFP* at day 10 only (chi-squared test $p<0.00001$). Significant differences between *GMR-Gal4/+;UAS-TDP43-M337/+* and *GMR-Gal4/UAS-TDP43-M337V; atx2 ΔcIDR-GFP/ atx2 ΔcIDR-GFP* at day 1, 10, 20 (chi-squared test $p<0.00001$), 30 (chi-squared test $p<0.006$) and day 40 (chi-squared test $p<0.02$).
